# Feasibility Using Telehealth for Planning Use of Extracorporeal Shockwave Therapy in a Sports Medicine Clinic

**DOI:** 10.3390/healthcare11111574

**Published:** 2023-05-27

**Authors:** Marissa J. Eckley, Connie Hsu, Adam S. Tenforde

**Affiliations:** Department of Physical Medicine and Rehabilitation, Spaulding Rehabilitation Hospital, Harvard Medical School, Boston, MA 02115, USA; meckley@mgh.harvard.edu (M.J.E.);

**Keywords:** telehealth, extracorporeal shockwave therapy, diagnostic agreement

## Abstract

(1) Background: The purpose of this study is to describe whether telehealth compared with in-person visits, led to a similar agreement of primary diagnosis reached at the time of procedure using extracorporeal shockwave therapy. (2) Methods: This retrospective study consisted of chart reviews of all new patients evaluated in a sports medicine clinic prior to performing extracorporeal shockwave therapy from April 2020 to March 2021. The primary outcome of the study was describing agreement in primary diagnosis at the time of evaluation (telehealth and in-person) and during the procedure using extracorporeal shockwave therapy. Logistic regression was utilized to identify patient characteristics that may predict agreement of diagnosis using telehealth. (3) Results: The chart review identified 166 patients (45 telehealth and 121 in-person) evaluated for extracorporeal shockwave therapy. Agreement of diagnosis was similar for patients evaluated using telehealth compared to in-person visits (84% vs. 92%, Χ^2^ = 1.90, *p* = 0.168). Agreement on diagnosis was more likely in patients who started shockwave within the 1 week of initial visit (OR = 8.27, 95% CI = 1.69–45.29), patients over age 60 (OR = 0.94, 95% CI = 0.90–0.99), and in patients without a history of osteoarthritis (OR = 14.00, 95% CI = 1.88–113.46). (4) Conclusions: Telehealth resulted in a similar agreement to in-person visits to identify a primary diagnosis for planning extracorporeal shockwave therapy. Telehealth may be a reasonable alternative to in-person visits for procedural planning of extracorporeal shockwave therapy.

## 1. Introduction

The rapid expansion of telehealth during the COVID-19 pandemic resulted from the need to identify safe methods to provide patient care. Studies have provided evidence that the expansion of telehealth resulted in similar delivery of care compared to traditional inpatient and outpatient settings [1,2]. Though initially utilized to limit contagion, recognized advantages of telehealth include decreased travel time, increased accessibility to care, and lower costs of telehealth compared to in-person visits for patients [3,4]. Additionally, both providers and patients have reported high satisfaction when using telehealth [5,6,7].

Telehealth continues to be used, given the logistical advantages. Hybrid models of healthcare delivery, which include a combination of telehealth and in-person visits, are becoming more common and are being utilized to increase access to care in underserved areas [8]. Therefore, it is important to characterize how telehealth can be used in place of in-person visits. For example, one limitation commonly cited with telehealth visits was being unable to perform a physical exam. However, a large number of studies and published guidelines outline virtual physical exam techniques and validity for use [9,10,11,12,13,14,15,16,17,18,19,20,21,22,23,24,25,26,27,28]. Telehealth has also been shown to allow for a similar evaluation of walking gait and the need for walking aids [29]. One study of patients evaluated virtually during COVID-19 found that subsequent in-person visits confirmed the treatment plan 84% of the time [30].

Few studies have evaluated procedural planning in sports medicine clinics. Prior studies suggest telehealth may have an acceptable agreement to guide planning in-person procedures [31,32,33]. Extracorporeal shockwave therapy (ESWT) generates energy in the form of sound and pressure waves that can promote tissue healing and disrupt pain [34,35]. ESWT has been shown to be effective in the treatment of tendon, bone, and joint pathologies [36,37,38,39,40,41,42,43,44,45,46,46,47,48,49,50,51,52]. Uniquely, ESWT utilizes the principle of clinical focusing, where the shockwave treatment is directed over the area of maximal pain [53]. This feedback during the application of ESWT may also help clarify the working diagnosis. To date, no studies have explored the use of telehealth compared with in-person visits in planning ESWT and reaching a similar diagnosis. The purpose of this study was to determine whether telehealth compared with in-person visits, would have a similar agreement in diagnosis reached during the procedure with ESWT. We hypothesized that telehealth would have a similar agreement as in-person visits for diagnosis at the time of ESWT.

## 2. Materials and Methods

This retrospective chart review was approved by the Institutional Review Board at Mass General Brigham. The electronic medical records of all new patients evaluated by the senior author to determine appropriateness for ESWT from March 2020 to April 2021 were reviewed by three authors (ME, CH, AST). Exclusion criteria included patients less than 18 years of age, those seen previously by the provider, underlying systemic neurologic and infectious conditions, and patients who did not receive ESWT. Clinic data, including conduction of the initial visit using telehealth or in person, diagnosis at the initial visit, diagnosis at the time of initial ESWT, prior visits with a sports medicine clinician in the Mass General Brigham network, imaging, and time between initial visit and initial ESWT were extracted. Diagnosis at time of initial visit was based on the primary diagnosis recorded in the assessment and plan of the senior author’s initial visit note (AST). Diagnosis at time of initial ESWT was based on the primary diagnosis recorded in the procedure note performed by the same physician (AST).

The senior author is double boarded in Physical Medicine and Rehabilitation and Sports Medicine. His clinic population includes patients self-referred or referred by other healthcare providers for musculoskeletal injuries. Prior to each visit, patients were requested to complete an intake form that was provided by paper for patients seen in-person or available through mail or by electronic delivery. This form was used in obtaining a comprehensive history, including date of onset of symptoms, past medical, family, and social history, 14-point review of systems, level of sports participation and goals of treatment, along with prior treatments to date. During the encounter, the physician (AST) obtained further clinical history and performed a physical examination or virtual exam for in-person and telehealth visits, respectively. The purpose of the initial visit whether conducted virtually or in-person, was to evaluate the patient and develop a treatment plan. Treatment options included pharmacologic, therapy-based (physical therapy, occupational therapy), non-surgical procedures, and consideration for surgical referrals as appropriate for each individual patient case. For some patients, the treatment plan included additional imaging prior to initiating ESWT. These visits also provide an opportunity to review expectations for ESWT with the patient.

The primary outcome variable was diagnostic agreement, as defined as agreement between the primary diagnosis at the initial visit (telehealth, in-person) and the primary diagnosis at initial ESWT. The agreement of the diagnoses was assessed during initial chart review by two authors (ME and CH) and then confirmed by the senior author and treating physician (AST). Demographic data, including age, gender, height, and weight, were also extracted from clinical records.

The primary outcome, agreement between the initial diagnosis using telehealth compared to an in-person visit, was assessed using the Chi-Square test. Logistic regression modeling was used to identify variables associated with increased agreement of initial diagnosis. Tested variables included age, gender, weight, height, availability of imaging at time of initial evaluation, availability of notes from other providers, history of osteoarthritis at the site, and time between initial visit and initial ESWT. Results of logistic regression are reported as Odds Ratios. Additional exploratory analysis was conducted to assess how predictors of diagnostic agreement differed between the telehealth and in-person populations. *p*-value < 0.05 was considered statistically significant. All analyses were conducted in R (version 4.2.1) [54].

## 3. Results

### 3.1. Patient Population

The initial review identified 250 patients who were evaluated by the senior author to determine appropriateness for ESWT from March 2020 to April 2021. Based on the following pre-determined criteria, patients were excluded from the study if: patients were less than 18 years of age (*n* = 6), there was no diagnosis in the initial visit note (*n* = 30), the patient was seen previously by the provider (*n* = 34), the primary diagnosis was neurologic in etiology (*n* = 12), and the patient did not receive ESWT (*n* = 2). The final cohort consisted of 166 patients included in the study, 45 who were initially evaluated via telehealth and 121 who were seen in-person. The patient populations were similar in terms of age, gender, BMI, anatomical location of the primary diagnosis, and whether they had been seen by another in-network physician prior to evaluation (Table 1). However, a higher percentage of patients completing in-person visits had an X-ray (XR) and/or a magnetic resonance imaging (MRI) prior to evaluation (*p* = 0.001) compared to those completing telehealth visits.

### 3.2. Factors Affecting Diagnostic Agreement

Initial evaluation by telehealth resulted in a similar diagnostic agreement (84.4%) compared to in-person visits (91.7%, X^2^ = 1.897, *p* = 0.168). Logistic regression modeling found a higher likelihood of diagnostic agreement among those patients who started ESWT within 1 week of the initial consultation (Table 2, OR = 8.27, 95% CI = 1.688–45.285), those patients without a history of osteoarthritis (OR = 13.998, 95% CI = 1.880–113.457) and patients greater than 60 years of age (OR = 0.943, 95% CI = 0.896–0.985). The presence of imaging (XR, MRI, or both), prior visit with an in-network physician, multiple diagnoses, gender, and BMI were included in the model but did not show a significant effect on the diagnostic agreement. The model was assessed by its receiver operating curve, which had an area under the curve of 0.84.

### 3.3. Exploratory Analysis of within Group Predictors

To further characterize factors associated with higher diagnostic agreement within each visit type, an exploratory analysis was completed for both telehealth and the in-person visits. Within the telehealth visits, a higher diagnostic agreement was achieved in those without a history of arthritis (Table 3, X^2^ = 8.459, *p* = 0.004) and in those with one site of injury (X^2^ = 3.965, *p* = 0.0464). This differed from the patients evaluated initially in-person. Within the in-person population, diagnostic agreement was more likely in patients who received ESWT within 1 week of the initial consultation (X^2^ = 6.812, *p* = 0.009). A diagnostic agreement was statistically different based on the anatomic region involved in patients initially evaluated in person (X^2^ = 15.420, *p* = 0.017) and trended towards a significant effect in patients initially evaluated via telehealth (X^2^ = 8.924, *p* = 0.063). Table 3 reviews the diagnostic agreement by anatomic location. The majority of patients were seen for pain localized to the foot or ankle (*n* = 97) with diagnoses including Achilles tendinopathy, extensor tendinopathy of the foot, flexor hallucis longus tendinopathy, peroneal tendinopathy, plantar fasciitis, posterior tibialis tendinopathy, calcaneal bone stress injury, metatarsal arthritis, metatarsal bone stress injury, and tibial sesamoid pain. Fewer were seen for hip pain (*n* = 61), which included diagnoses of hip impingement, gluteal tendinopathy, greater trochanteric pain syndrome, hamstring tendinopathy, iliopsoas tendinopathy, iliotibial band syndrome, piriformis syndrome, and proximal quadriceps tendinopathy. The diagnostic agreement was higher in those presenting with foot or ankle pain compared to hip pain (96% vs. 87%, *p* = 0.038). When stratified by anatomic region, the rate of diagnostic agreement was relatively similar whether evaluated via telehealth or in-person. Though not reaching the threshold of statistical significance, a lower level of diagnostic agreement was observed in females compared to males within the telehealth cohort (76% vs. 95%, X^2^ = 3.053, *p* = 0.081). This trend was not seen in the patients initially evaluated in-person (94% vs. 88%, X^2^ = 1.468, *p* = 0.480). It is worth noting that 13 of the 27 females who were evaluated via telehealth presented with hip pain.

## 4. Discussion

The purpose of this study was to evaluate whether telehealth can be used to determine a diagnosis in planning ESWT compared to in-person visits. We observed that telehealth and in-person visits resulted in a similar agreement in diagnosis determined at subsequent times of ESWT. These results are consistent with the growing body of literature that supports the validity of virtual physical exam techniques [9,10,11,12,13,14,15,16,17,18,19,20,21,22,23,24,25,26,27,28] and represents a functional application of those techniques. While it is unlikely that a telehealth physical exam is equivalent to an in-person exam, our findings suggest the initial visits (telehealth and in-person) resulted in sufficient information to generate an appropriate working diagnosis for ESWT. Some functional maneuvers (such as a calf raise for Achilles tendon integrity) may be used during telehealth visits to replace examiner-dependent maneuvers (such as the Thompson test) to determine whether an injury would be appropriate to treat with ESWT or require further diagnostic testing or surgical consultation.

In patients evaluated using telehealth, those with a history of osteoarthritis and who presented with multiple sites of pain had associated lower diagnostic agreement at the time of ESWT. These findings suggest a subset of patients may benefit from in-person evaluation when planning ESWT, particularly those with osteoarthritis and/or multiple sites of pain. While we cannot determine the exact reason for these findings, we suspect lower diagnostic agreement is related to limitations in the telehealth physical examination. For example, osteoarthritis is a condition that is often evaluated with maneuvers that rely on a passive range of motion (such as performing an internal range of motion or log roll testing of the hip), which is more challenging to have a patient self-perform.

Our study also identified the following two patient characteristics affecting diagnostic agreement when planning ESWT: age above or below 60 years old and the anatomic region of pain. There is limited literature on the effect of age on diagnostic accuracy; one study looking at the diagnostic accuracy of shoulder physical exam maneuvers found higher accuracy among patients over 39 years of age [55]. In our study, diagnostic agreement was significantly higher in those patients presenting with foot/ankle pain compared to hip pain. A systematic review and meta-analysis found weak diagnostic properties for common physical exam maneuvers for hip pain [56]. A separate systematic review and meta-analysis identified clinical history as more relevant than physical examination in the diagnosis of hip pain [57]. Given that other anatomic regions made up less than 10% of our sample, it is difficult to draw conclusions about other anatomic regions. However, it is notable that the only patient evaluated via telehealth for an upper extremity pathology had a change in diagnosis, given that a similar study of virtual consultation for orthopedic surgery found decreased accuracy for the upper compared to lower extremities [32].

Further, there was a trend towards reduced agreement on diagnosis for females evaluated via telehealth, though not reaching a threshold of statistical significance. Notably, hip pain was the presenting complaint of 48% of females evaluated via telehealth compared to 31% evaluated in-person. Hip pain is associated with decreased agreement and may partially explain our finding of the lower diagnostic agreement for females. However, prior work has observed gender influencing telehealth utilization [58,59] as well as the length of telehealth visits and physician prescribing patterns [60]. Further, a prior study evaluating the feasibility of telehealth for the planning of interventional spine procedures saw a trend toward higher diagnostic agreement in male patients [31]. Further investigation is necessary to understand whether gender influences how telehealth can be used for procedure planning and methods to optimize use for all genders.

Our findings support the expanded use of telehealth for procedural planning within a sports medicine clinic. A study of 303 orthopedic patients who were initially seen virtually for preoperative planning and then seen in-person prior to the operation found that only 4% of procedural plans changed during the subsequent in-person evaluation [32]. A review of preoperative planning for spine surgery via telehealth has shown that surgical plans are rarely changed [31], have similar diagnostic accuracy to in-person evaluation [61,62,63], and resulted in similar postoperative outcomes [63,64]. These findings are not unique to musculoskeletal medicine. For example, A study looking at preoperative planning of minimally invasive urologic procedures found no change in the procedural plan of 45 patients evaluated only virtually before the procedure with similar perioperative outcomes as the control group [33].

This is the first study to date looking at telehealth compared to in-person visits for the planning of ESWT. Patients attending telehealth follow-up visits after receiving ESWT for Achilles tendinopathy or plantar fasciitis have previously been shown to have similar functional outcomes compared to those seen in-person for follow-up care [65]. The expansion of telehealth services allows for increased accessibility to care with decreased travel time and a lower cost for patients and society compared to traditional in-person visits [3,4]. This is particularly relevant within the area of specialty care and procedures, including ESWT.

There are limitations to this study. The sample size is relatively small, and there is an unequal distribution between the two groups, with fewer patients (27%) completing telehealth visits. Due to the retrospective nature of the study, we had to rely on chart review for documentation. Additionally, patient charts were reviewed by only one provider who is board certified in Physical Medicine and Rehabilitation and Sports Medicine; therefore, this study may not be generalizable to all providers. Obtaining the history and physical exam maneuvers performed was at the discretion of the provider without a defined methodology that would be established for a formal research investigation. Furthermore, the setting of the COVID-19 pandemic influenced the use of telehealth, and resources varied in the healthcare setting over the course of this study (e.g., access to advanced imaging procedures) may have affected the results.

We also note there is selection bias within our patient population that limits the generalizability of our study. The procedure ESWT has out-of-pocket costs, which are not covered by insurance, introducing a socioeconomic bias within our population that may not be generalizable to other procedures. There may be further selection bias in those patients who elected to complete a telehealth visit, including access to technology to complete these visits.

Future directions include reviewing long-term outcomes in those patients who were initially evaluated virtually compared to in-person. Additionally, a prospective study could provide a standardized approach to the history and physical exam, which may provide a more specific methodology in the planning of ESWT. Future studies would ideally include a large patient population pulled from multiple geographic regions with multiple providers.

## 5. Conclusions

The logistical advantages of telehealth and similar diagnostic agreement suggest the use of telehealth may be considered for procedural planning of ESWT. Patients with a history of osteoarthritis and/or more than one complaint may benefit from an in-person visit. Exploring the influence of gender and anatomy to improve diagnostic accuracy could be explored to understand how to optimize the use of telehealth.

## Figures and Tables

**Table 1:** Demographic and clinical characteristics of included patients.

	All Patients (*n* = 166)	Telehealth (*n* = 45)	In-Person (*n* = 121)	Group Difference
Age, mean (SD)	43.7 (16.3)	40.6 (15.8)	44.8 (17.2)	*p* = 0.161
Gender, %				
Female	56.7	55.6	57	Χ^2^ = 0.425
Male	42.8	44.4	42.1	*p =* 0.809
Nonbinary	0.6	0	0.8	
BMI, %				
Underweight	2.4	2.2	2.5	
Normal Weight	60.2	60	60.3	Χ^2^ = 0.463
Overweight	27.7	24.4	28.9	*p =* 0.927
Obesity	7.2	8.9	6.6	
Not Recorded	2.4	4.4	1.7	
Location of Pathology, %				
Shoulder	2.6	2	2.8	
Elbow/Forearm	2.6	0	3.5	
Wrist/Hand	0.5	0	0.7	Χ^2^ = 6.166
Back	2.6	2	2.8	*p* = 0.405
Hip/Thigh	33	42.9	29.6	
Knee/Leg	9.4	14.3	7.7	
Ankle/Foot	49.2	38.7	52.8	
Etiology of Pathology, %				
Bone/Joint	20.4	22.4	19.7	Χ^2^ = 0.167
Muscle/Tendon	79.6	77.6	80.3	*p = 0.682*
Imaging Available, %				
None	50.6	73.3	42.1	Χ^2^ = 16.607
XR only	12.7	4.4	15.7	***p =* 0.001**
MRI only	25.9	22.2	27.3	
Both	10.8	0	14.9	
Prior Visit with MGB Sports Medicine Attending, %				Χ^2^ = 0.010
	28.3	28.9	28.1	*p* = 0.920

Bolded values were statistically significant at a threshold of *p* < 0.05. SD = standard deviation, BMI = body mass index, XR = X-ray, MRI = magnetic resonance imaging, MGB = Mass General Brigham.

**Table 2:** Binomial logistic regression results of demographic and clinical characteristics for prediction of diagnostic agreement.

	B	SE	Z Score	*p*	OR	95% CI
Intercept	−1.655	1.163	−1.423	0.155	0.191	0.017–1.731
Telehealth	−0.465	0.839	−0.554	0.580	0.628	0.120–3.352
XR	−0.574	1.229	−0.047	0.963	0.944	0.043–8.256
MRI	1.184	0.701	1.688	0.0914	3.27	0.841–13.749
XR & MRI	1.794	1.105	1.624	0.104	6.015	0.598–54.127
Prior MGB Sports Medicine Visit	0.632	0.662	0.854	0.340	1.881	0.501–7.003
More than 1 week before Shockwave	**2.107**	**0.825**	**2.555**	**0.011**	**8.27**	**1.688–45.285**
History of Arthritis	**2.639**	**1.017**	**2.597**	**0.009**	**13.998**	**1.880–113.457**
More than 1 diagnosis	1.110	0.751	1.478	0.140	3.034	0.648–13.229
Age ≥ 60 years old	**−0.583**	**0.0239**	**−2.437**	**0.0148**	**0.943**	**0.896–0.985**
Female	−0.207	0.638	−0.324	0.746	0.813	0.231–2.926
BMI ≥ 25 kg/m^2^	−0.607	0.716	−0.849	0.396	0.545	0.119–2.091

Bolded values were statistically significant at a threshold of *p* < 0.05. History of Arthritis refers to a prior diagnosis of osteoarthritis. XR = X-ray, MRI = magnetic resonance imaging, MGB = Mass General Brigham, BMI = body mass index.

**Table 3:** Association of demographic and clinical characteristics to diagnostic agreement among patients evaluated virtually compared to in-person.

	Telehealth				In-Person			
	Per Patient (*n* = 45)		Per Diagnosis (*n* = 49)		Per Patient (*n* = 121)		Per Diagnosis (*n* = 142)	
	Χ^2^	*p*	Χ^2^	*p*	Χ^2^	*p*	Χ^2^	*p*
Imaging	0.523	0.770	0.474	0.788	3.751	0.290	4.027	0.258
Prior MGB Sports Medicine Visit	0.000	0.984	0.062	0.804	0.764	0.382	0.849	0.357
More than 1 week before Shockwave	2.072	0.150	1.838	0.175	**6.812**	**0.009**	**7.256**	**0.007**
Hx of Arthritis	**8.459**	**0.004**	**7.122**	**0.008**	0.771	0.379	0.728	0.394
More than 1 Diagnosis	**3.965**	**0.0464**			0.095	0.757		
Age ≥ 60 years old	0.0102	0.920	0.0249	0.875	0.756	0.385	0.799	0.371
Sex, Female	3.053	0.081	3.093	0.079	1.468	0.480	1.581	0.454
BMI Category	3.033	0.219	2.892	0.235	0.912	0.822	0.869	0.833
Pathology: Bone/Joint vs. Muscle/Tendon			0.176	0.675			1.897	0.168
Anatomic Region			8.924	0.063			**15.420**	**0.0172**

Bolded values were statistically significant at a threshold of *p* < 0.05. History of Arthritis refers to a prior diagnosis of osteoarthritis. MGB = Mass General Brigham, Hx = history, Dx = diagnosis, BMI = body mass index.

## Data Availability

Data are available with reasonable request.
